# Multifunctional Flexible Sensor Based on Laser-Induced Graphene

**DOI:** 10.3390/s19163477

**Published:** 2019-08-09

**Authors:** Tao Han, Anindya Nag, Roy B. V. B. Simorangkir, Nasrin Afsarimanesh, Hangrui Liu, Subhas Chandra Mukhopadhyay, Yongzhao Xu, Maxim Zhadobov, Ronan Sauleau

**Affiliations:** 1 DGUT-CNAM Institute, Dongguan University of Technology, Dongguan 523106, China; 2 University of Rennes 1, UMR CNRS 6164, Institut d’Electronique et de Télécommunications of Rennes (IETR), F-35000 Rennes, France; 3 School of Engineering, Macquarie University, Sydney, NSW 2109, Australia; 4 Department of Astronomy and Physics, Macquarie University, Sydney, NSW 2109, Australia

**Keywords:** capacitive sensors, electrochemical sensing, flexible sensors, interdigital, laser-induced graphene, strain sensing, wearable sensors

## Abstract

The paper presents the design and fabrication of a low-cost and easy-to-fabricate laser-induced graphene sensor together with its implementation for multi-sensing applications. Laser-irradiation of commercial polymer film was applied for photo-thermal generation of graphene. The graphene patterned in an interdigitated shape was transferred onto Kapton sticky tape to form the electrodes of a capacitive sensor. The functionality of the sensor was validated by employing them in electrochemical and strain-sensing scenarios. Impedance spectroscopy was applied to investigate the response of the sensor. For the electrochemical sensing, different concentrations of sodium sulfate were prepared, and the fabricated sensor was used to detect the concentration differences. For the strain sensing, the sensor was deployed for monitoring of human joint movements and tactile sensing. The promising sensing results validating the applicability of the fabricated sensor for multiple sensing purposes are presented.

## 1. Introduction

The deployment of flexible sensors for ubiquitous sensing has been receiving ever-growing research interest in recent times. Different kinds of flexible sensors have been developed using a wide range of processing materials and fabrication techniques [1,2,3,4,5,6,7,8,9,10,11]. The responses of the fabricated prototypes have also been characterized for various sensing scenarios, quantified through various sensing parameters, from sensitivity and input power to the change of capacitance, resistance, and impedance. Graphene, since its discovery [12], has been one of the emerging conductive materials for realization of flexible sensors for various sensing applications [2,4]. Some of the advantages of graphene lie in its excellent electrical conductivity, high mechanical flexibility, and adaptability towards chemical and biological sensors.

Researchers have developed various graphene-based sensors exploiting the properties of pure and composite-based graphene [4,13,14,15,16,17,18,19,20,21,22]. Although a significant amount of work has been done on graphene sensors till now, there are still some issues that need to be addressed. Firstly, the production of graphene in general is an expensive process involving delicate instruments which require particular expertise to operate. It also includes multiple steps which increase the probability of forming low-quality graphene. For example, Chemical Vapor Deposition (CVD) being one of the most favored ways for mass production of graphene has certain disadvantages of the toxic gaseous byproducts, the use of harsh chemicals and the needs of high amount of energy [23,24]. Other techniques such as mechanical exfoliation and Hummer’s method also have other disadvantages which include a low output yield and a low purity of graphene due to the presence of exfoliated agent remnants and different kinds of defects e.g., atomic, ripples and microscopic corrugation [25]. These bring the need to look for alternate approaches to form graphene for sensor realization.

The second issue currently faced by the microelectronics industry is the lack of multifunctional sensors for monitoring purposes. Even though some researchers have recently worked on the development of graphene-based multifunctional sensors [18,19,20,21,22], there are certain limitations associated with them demanding for new sensing prototypes. For example, the performance of graphene-based Field Effect Transistors (FET) for sensing purposes shows drastic degradation in response at higher frequencies and poor linearity [22]. In some cases, the use of reduced graphene oxide (rGo) as the conductive material to develop the sensor degrades their performance due to the presence of impurity [20]. Also, the drop-casting of the rGO film would create an uneven layer which would even the performance the sensor. For certain sensors, even though the use of nanocomposites to develop the electrodes does provide mechanical advantages, the electrical conductivity gets compensated to a large extent which decreases their resultant sensitivities [19,21]. Therefore, the fabrication and implementation of multifunctional graphene sensors circumventing the above-mentioned issues are necessary.

In this paper, to address the aforementioned issues, we present a low-cost and easy-to-fabricate graphene-based sensor, together with its potential for multiple applications. We opted for a laser-induction process on commercial polyimide film to generate graphene, which was then transferred onto sticky Kapton polyimide tape through manual pressure by hand. The graphene layer is subsequently used as electrodes of the sensor prototype, whereas the Kapton tape is used as the substrate. We used the class of polyimide as the sensor substrate considering its advantages of high flexibility, biocompatibility, excellent chemical resistance and high tolerance strength [26]. The novelty of this paper lies in the fabrication process and subsequent implementation of laser-induced graphene sensor. Firstly, the proposed fabrication approach solves the detachment issue of the graphene layer that is likely to happen in the case where the graphene formed on the source materials (e.g., polyimide film or recently natural woods or leaves), were directly used as the sensor electrodes, as commonly demonstrated in a lot of work [27,28,29,30,31,32,33]. At the same time, our proposed approach is simpler than the formulation of composite structures using elastomer or any other classes of material as shown in [34,35,36,37], which were proposed as alternative solutions for fabricating laser-induced graphene-based sensors. Secondly, the use of laser-induced graphene has been mainly done for developing micro-supercapacitors or energy-harvesting devices [34,35,38,39,40,41,42], whereas in this paper, we highlighted the use of such material for development of interdigitated capacitive-based sensor with multi-sensing capability.

To demonstrate its multi-functionality, we implemented the sensor in electrochemical and strain-sensing scenarios. For the electrochemical sensing, as a proof of concept, the sensor was used to test sulfate solutions at different concentrations. The motivation behind this idea is to emphasize the sensing of sulfate concentrations in the soil at a specific instant. As per literature [43], the sulfate concentrations in the soil vary between 1000 ppm and 10,000 ppm with a risk level being associated with any concentration above 3000 ppm. The presence of excess amount of sulfate in the soil can be detrimental to existing flora and fauna causing stunted growth, killing aquatic life via seeping of acid sulfate soils in water and mental impairment caused by drinking the acidic sulfate water. On the other hand, for the strain sensing, the sensor was opted for monitoring of human joint movements and tactile sensing. The idea behind these two scenarios is to investigate the applicability of the sensor as wearable sensing prototypes for enabling the accurate and efficient real-time data acquisition in the emerging human-activity monitoring and personalized healthcare systems [44].

The remaining parts of the paper have been divided into five sections. In Section 2 and Section 3 we describe the fabrication process and the working principle of the sensor, respectively. Following that, the results and discussion regarding the implementation of the sensor for the two applications mentioned above are given in Section 4. Finally, the conclusion is drawn in Section 5.

## 2. Fabrication of the Graphene Sensor

**Figure 1:**
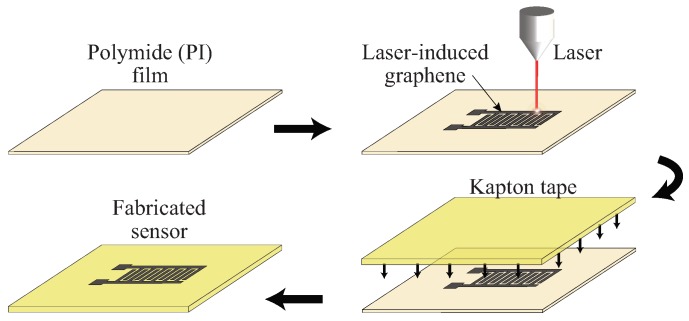
Schematic diagram illustrating the fabrication process of the laser-induced graphene sensor.

The fabrication process of the laser-induced graphene sensor is shown in Figure 1. A capacitive sensor with two electrodes, each with six interdigitated fingers, was designed to demonstrate the concept. Commercial polyimide film (Zibo Zhongnan Plastics Co., Ltd.), with a thickness of 0.025 mm was used as the processing material to form the laser-induced graphene. An OLS 6.75 CO${}_{2}$ laser system with a laser spot diameter of 150 μm was used to perform the laser-induction process upon the film attached on a glass substrate with a tape. The laser-irradiation of the polyimide film caused the photo-thermal generation of the graphene as a result of local heating of the film. The carbon atoms being bonded with oxygen (C–O, C=O) and nitrogen (C–N) atoms via sp${}^{3}$ and sp${}^{2}$ hybridization were broken down and rearranged to form several layers of sp${}^{2}$ hybridized carbon atoms of graphene [27]. The irradiation process was obtained by optimizing the laser parameters, including power, speed, and *z*-axis of the laser system. Power refers to the magnitude of the energy on which the laser nozzle operates. Speed (m/min) refers to the rate of movement of the laser nozzle over the sample in *x*-*y* directions. *z*-axis (mm) defines the focal point of the laser on the sample. The optimized values of these parameters were: 7 W of power, 70 m/min of speed, and 1 mm of *z*-axis.

Next, the patterned graphene was meticulously transferred to commercial Kapton sticky tape (thickness of 0.1 mm), used as the substrate of the sensor. The process was done by placing the tape above the formed graphene and applying manual pressure over it, starting from the bonding pads and moving towards the electrode fingers. The graphene formed on the surface of polyimide film is very easy to shear off. The transfer of graphene to the sticky surface of Kapton tape therefore significantly improves the mechanical robustness of a laser-induced graphene-based sensor. Under a Scanning Electron Microscope (SEM), we also observed that the manual pressure applied during the transfer process leads to morphological changes of the graphene network, which include a more regular porosity, seemingly reduced thickness, and better interconnection between multilayer graphene flakes. The photo-thermal formation of graphene was not done directly on the Kapton tape, despite being made of polyimide, due to its stickiness that would cause an immediate agglomeration of the induced graphene, thus tampering the design of the electrodes. Moreover, Kapton tape provides a better physical robustness to the sensor than a thin polyimide film.

**Figure 2:**
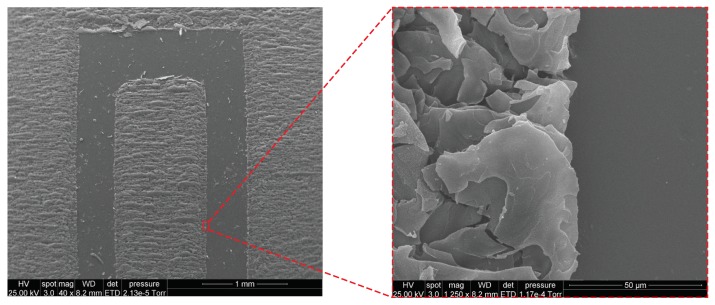
Morphology of the fabricated graphene sensor taken under SEM.

**Figure 3:**
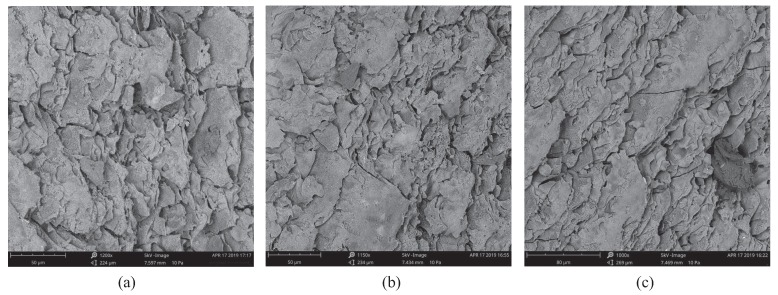
SEM images of the graphene morphology: (**a**) before tests, (**b**) after repetitive electrochemical sensing tests, and (**c**) after repetitive mechanical sensing tests. During the electrochemical test, the sensor was immersed in five samples with different concentrations and the process was repeated three times. During the mechanical test, repetitive bending-releasing movements were applied to the sensor for 25 min. The details are given in Section 4.

The SEM images of the top view of the transferred graphene on Kapton tape are shown in Figure 2. The images were taken with a PHENOM XL Benchtop SEM. It can be seen from these images the clarity of the transferred graphene over the Kapton tape. The graphene particles as seen from the 1250× magnified view are distributed quite uniformly, well connected, which negates any deterioration in the electrical conductivity of the electrodes. The graphene flakes show an ultra-polycrystalline feature, indicating the existence of an abundance of grain boundaries composed of pentagon and heptagon pairs, which account for the porous structure of graphene [27]. Such structure offers a higher surface area and good sensitivity for sensing purposes [29]. The sticky nature of the Kapton tape kept the graphene powder intact, even after performing experiments with the sensor. To validate this, we once again observed under SEM, the graphene morphology of the sensor after complete cycles of repetitive electrochemical and mechanical sensing tests elaborated in Section 4. The comparison between the graphene morphology of the sensor before and after the tests is given in Figure 3. As can be seen, there is no significant morphological difference between them. It is later shown that the sensing performance was also relatively stable throughout the repetitive tests. This demonstrates that the proposed fabrication approach, i.e., the use of sticky Kapton tape and the manual pressure during the transfer of graphene, results in a firm attachment of the graphene powder, to survive the liquid immersion and physical deformation.

**Figure 4:**
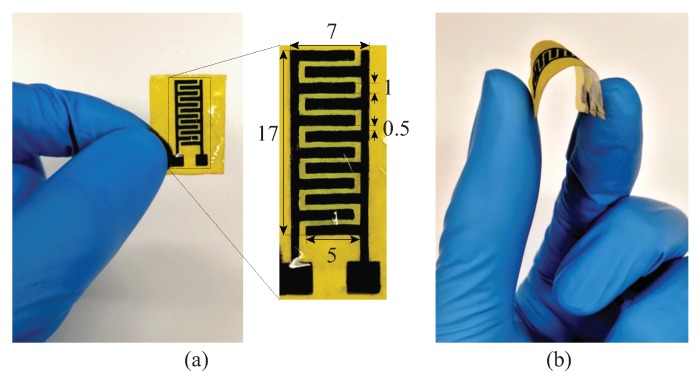
(**a**) Fabricated sensor with detailed design (all dimensions are in millimeters). (**b**) The bending view showing the flexibility of the sensor.

Figure 4a shows the fabricated graphene sensor along with its design parameters obtained based on [45,46], considering the response of the capacitive sensor. The sensing area of the sensor was 119 mm${}^{2}$ and each interdigitated finger has a length and width of 5 and 1 mm, respectively. The gap between two fingers is maintained to be 0.5 mm. Such dimensions were found to be large enough for employment in both electrochemical and strain-sensing scenarios applied in this work. Apart from the electrical properties imparted by the interdigitated graphene electrodes, the sensor was also highly bendable, as shown in Figure 4b. These two inherent properties of the sensor increase their potential to be used for electrochemical and strain-sensing purposes.

## 3. Working Principle of the Sensor

**Figure 5:**
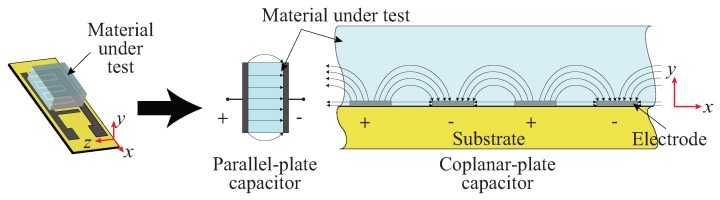
Illustration of the electrodynamics of a capacitive sensor.

One of the operating principles of the interdigitated capacitive sensor used in this work is based on the electrodynamics of two parallel plate capacitor as shown in Figure 5. Once an AC voltage is applied between the positive and negative electrodes, electric fields are generated. If any materials are in vicinity, the generated electric fields penetrate through the materials, which in turn leads to the changes particularly in the capacitive reactance of the sensor, and hence its impedance. Therefore, by measuring the changes in the impedance, the electrochemical sensing behavior can be observed. It is also worth noting that due to the coplanar nature of the sensor, there are more prominent fringing field lines in addition to the direct field lines between the fingers as compared to the parallel plate type sensor. This makes the coplanar type capacitive sensor more sensitive to the changes in the electrical properties of surrounding medium.

**Figure 6:**
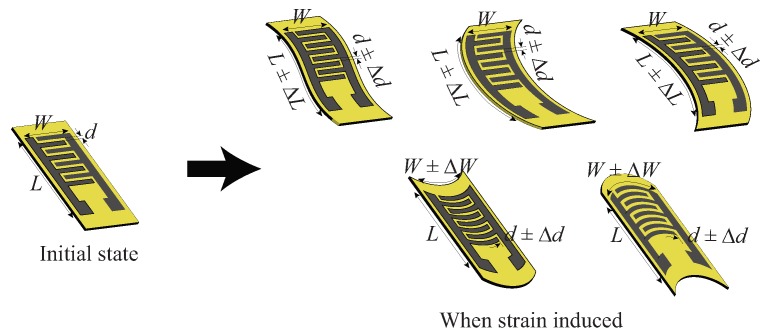
Illustration of the induced strain affecting the overall dimensions of the sensor.

Another sensing mechanism of the sensor heavily relies on the dependency between the sensor geometry (length (*L*), width (*W*), and gap between interdigitated fingers (*d*)) and its electrical properties. Due to its flexible nature, any strain induced on the sensor leads to the change in its overall dimensions (Δd, ΔL, ΔW) as compared to its initial state. This is illustrated in Figure 6 showing a wide range of deformations possibly happen to the sensor. For instance, when the sensor is uniaxially bent, it is elongated in the length or width direction of the sensor which consequently changes the gap between the fingers in either direction, and hence the overall capacitance of the sensor. Such deformations concurrently affect the distribution of the graphene over the substrate, which contributes to the change in the resistance of the sensor. Consequently, the total impedance (*Z*) of the sensor changes, through which, therefore, strain can be sensed and quantified. In this work, we reported the sensor response in terms of magnitude of the complex total impedance (|Z|).

## 4. Potential Applications of the Laser-Induced Graphene Sensor

Based on two sensing principles described above, the sensor developed in this work is proposed for two applications, electrochemical sensing, and strain sensing. The applicability of the sensor for both applications was verified through two scenarios. Firstly, the sensor was applied to monitor different sulfate concentrations in water samples. Secondly, it was implemented to sense the strains induced by the movements of human joints and the touches of body part (tactile sensing). The responses of the sensor were analyzed through impedance spectroscopy. The experiment setup consisted of an LCR meter HIOKI IM3536 connected to a computer via a USB cable for data acquisition and storage. A Hioki 9140 4-terminal test probe with crocodile clip termination was used to connect the LCR meter with the sensor. All the experiments were operated in laboratory conditions at fixed temperature of 25 ${}^{\circ }$C and humidity of 50% RH.

### 4.1. Electrochemical Sensing of Sulfate in Water Samples

**Figure 7:**
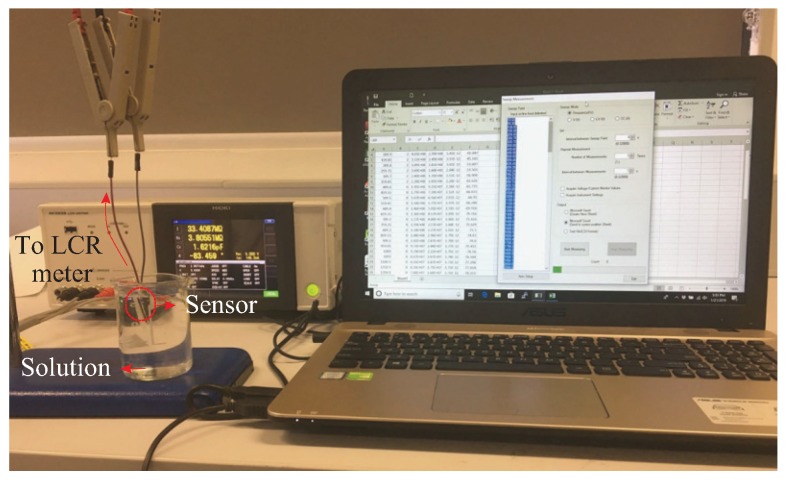
Experimental setup for testing of sulfate samples at different concentrations.

Figure 7 shows the experimental setup used for the measurement of the sulfate concentrations. Two wires were connected to the bonding pads of the electrodes with conductive silver epoxy. The sensor was carefully dipped inside the sample up to the level of the bonding pads and its impedance response was measured. To restrict the movements of the sensor during the experiment, it was attached onto an acrylic board with tape before dipping it into the sample. Five different samples with concentration levels ranging between 1 ppm and 10,000 ppm were used. An input voltage of 1 V RMS with frequency swept from 10 Hz to 4000 Hz was applied to the sensor. The immersion of the sensor during data collection was normally in a matter of seconds. To validate the consistency of the sensor responses towards the measured concentrations, the experiment was repeated three times for each concentration. A gap of 5 min was given between each cycle, where the sensor was washed and dried before the next experiment.

The preparation of the five samples was done through a serial dilution process. Anhydrous sodium sulfate (Alfa Aesar, CAS: 7757-82-6) and deionized (DI) water were used as the solute and solvent, respectively. An initial solution of 10,000 ppm was formed by mixing 1 g sodium sulfate with 100 mL DI water to create the first sample. After the experiment with the first sample, 10 mL of this sample was pipetted to 90 mL of DI water to form the sample with the second concentration of 1000 ppm. This process was repeated until the fifth sample with a sulfate concentration of 1 ppm.

**Figure 8:**
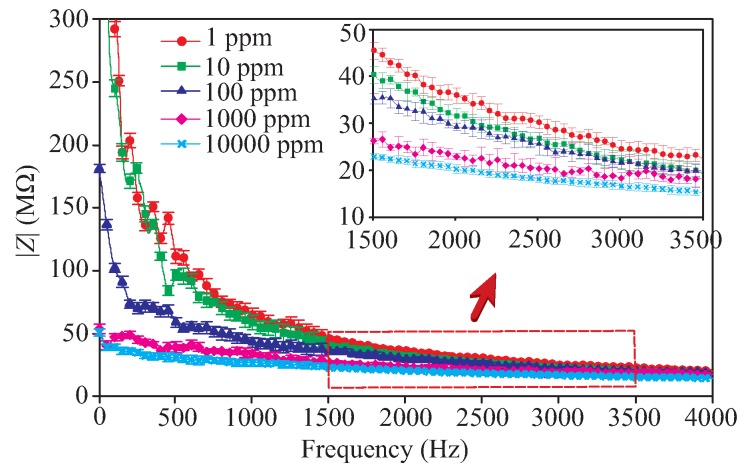
Impedance response of the sensor for different concentrations of sulfate.

Figure 8 shows the relation between the sensor impedance with the sulfate concentration over the observed frequency range. averaged over the three sets of measured results obtained for each concentration. Once the conductive electrodes are immersed into the sulfate solution, and a voltage is applied, two layers of polarized ions are generated at the interfaces of the electrodes. One layer is formed within the conductive electrodes whereas the other layer with an opposite polarity is distributed in the solution. The latter is composed of solvated ions that are attracted towards the polarized electrodes. Both layers are typically separated by a single layer of hydrated molecules acting like a dielectric in a conventional capacitor. The physical and chemical interactions between the ions in the electrodes and those in the solution cause a nonlinear charge distribution, which results in a double layer capacitance phenomenon [47]. Together with the direct capacitive coupling between the interdigitated electrodes [47], this phenomenon leads to the impedance behavior shown in Figure 8. The sensor impedance is high when the concentration of the sulfate is low and decreases as the concentration level increases. This is due to the decrease in the solution resistance and the increase of the double layer capacitance along with the rise of the ion concentration in the solution [47]. It can also be seen that the sensor seems to be more sensitive in the low concentration range as a result of the unsaturated distribution of the solvated ions (i.e., Na${}^{+}$ cations/SO${}_{4}^{2-}$ anions) at the electrodes’ interfaces [11],and hence their electrostatic interaction with the polarized ions within the electrodes.

**Figure 9:**
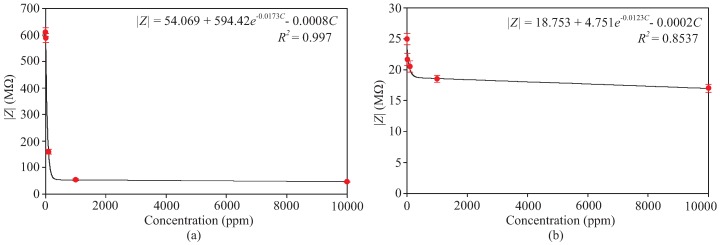
Sensitivity of the graphene sensor towards the sulfate concentrations at (**a**) 28 and (**b**) 3500 Hz.

It was also reported in [47] that particularly in lower frequency, the sensor impedance is highly dominated by the concentration dependent double layer capacitance, until its impedance becomes lower than the resistance of the electrolyte and the sensor impedance becomes relatively independent of frequency [47]. This explains the trend of the sensor impedance shown in Figure 8 as well as Figure 9, where we selected two different points (i.e., 28 and 3500 Hz) from the range of frequency observation and depicted their impedance with respect to the ion concentrations. The latter is presented to highlight further the electrochemical sensitivity of the sensor at the low and high-frequency regions. As shown, the decreasing rate of the impedance changes around the concentration range of 80 to 200 ppm which seems to be the transition point mentioned before (i.e., where the sensor impedance is lower than the solution resistance). Considering this, we believe that it is most appropriate to express the sensitivity of the sensor into two values, each representing the sensitivity of the region before and after the transition point, which we later refer to the sensitivity values of low (1 to 100 ppm) and high (100 to 10,000 ppm) concentrations, respectively. In the low-frequency region, the sensitivities of 0.8 and 0.18 W/ppm were obtained for low and high concentration ranges, respectively, while in the high-frequency region, they were found to be 0.065 and 0.035 W/ppm for low and high concentration ranges, respectively. Overall the results in Figure 8 and Figure 9 clearly show that the developed sensor can be used for effective differentiation of the sulfate concentration and at the same time imply the best frequency operation for this sensing purpose, i.e., approximately within 10 to 500 Hz range.

**Figure 10:**
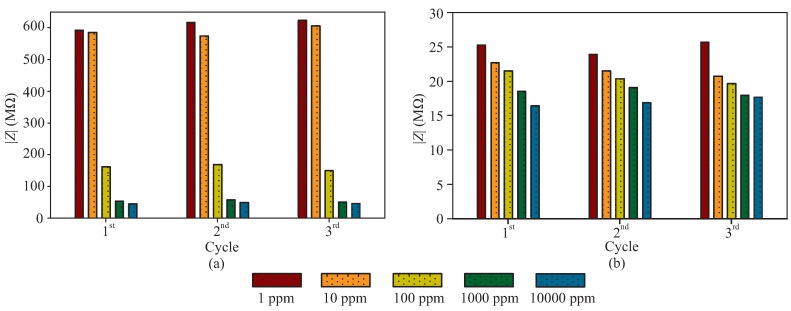
The repetitive electrochemical response of the sensor at (**a**) 28 and (**b**) 3500 Hz.

To further highlight the consistency of the sensor responses implied in Figure 8 and Figure 9, we also depict in Figure 10a,b the responses of the sensor for each measurement cycle at the selected frequencies of 28 and 3500 Hz. As can be seen, the responses of the sensor for each cycle were very close to each other. The decreasing trend in the change of the impedance is also maintained for each cycle. The calculated relative standard deviations of the repetitive tests for five different concentrations (i.e., from 1 to 10,000 ppm) are 2.72%, 2.78%, 5.99%, 6.55%, and 3.76% at 28 Hz and 3.72%, 4.63%, 4.39%, 3.15%, and 3.67% at 3500 Hz, validating the similarity of the results of the three cycles. With the sensor’s flexibility, quick response time, sensitivity, and biocompatibility, it can therefore be implied that such sensor would be a great candidate to be applied in wearable environment for electrochemical sensing of different analytes on the human body.

### 4.2. Sensing of Strains Induced by Human Body Movements and Touches

**Figure 11:**
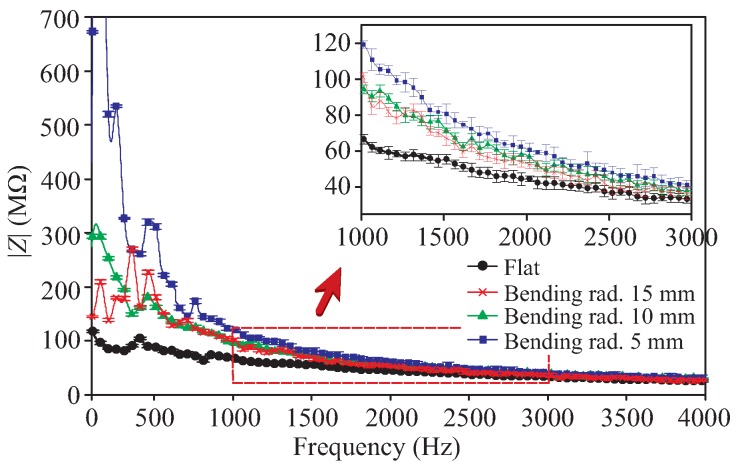
Response of the sensor for different bending radii of curvature.

Prior to applying the sensor for sensing of human body-induced strains, we measured the sensor impedance as a function of frequency. In this case, we situated the sensor in four different conditions, i.e., flat and bent over three different curvatures. The aim was to briefly investigate the response of the sensor towards the change in its physical dimensions. The response is depicted in Figure 11 averaged over the results of four measurements taken per condition. As can be seen, the changes in the impedance were obvious, suggesting a potential for strain sensing. In comparison to the result when the sensor is flat, the impedance of the sensor increases when the bending radius decreases. This is predominantly due to the increase in the gap between the interdigitated fingers, leading to the decrease of capacitance. A decreasing trend over frequency can be seen in the impedance values as well as the sensitivity of the impedance. Even though the sensor seems to be more sensitive in the low-frequency region (<1000 Hz), the change in the impedance is as not as stable as that of the upper frequency. For this reason, it was decided to apply 1000 Hz AC signal for the rest of the measurements in this section.

**Figure 12:**
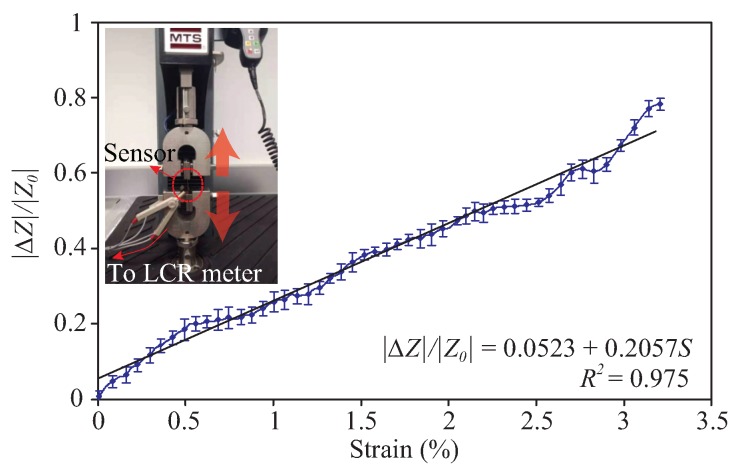
Change in sensor impedance with respect to different strain values applied in the direction illustrated in the inset. The impedance change (|ΔZ|) is shown relative to the impedance when no strain induced ($|Z_{0}|$).

We further quantified the flexibility and the impedance characteristic of the sensor as a function of strain through a tensile-strength measurement by means of EXCEED E42 MTS Universal Test Device. The sensor was clamped firmly and the strain was applied to the sensor by moving the crosshead with a velocity of 0.2 mm/s in a perpendicular direction to the interdigitated fingers (see inset of Figure 12). The obtained stress-strain response of the fabricated sensor shows a linear elastic region until a strain value of 3.3% before finally failure at a strain of approximately 8%. The calculated Young’s modulus of the sensor was found to be 0.23 GPa.

The impedance response of the sensor corresponding to the level of the strain applied was also measured and shown in Figure 12. The measurement setup can be seen in the inset. For connection to the LCR meter, two thin copper wires were attached to the electrode pads by means of conductive silver epoxy. For this purpose, an alternating signal of 1 V RMS and 1000 Hz frequency was applied to the electrodes. As can be seen in Figure 12, the sensor responds quite linearly to the applied strain within a strain range of 0% to 3.3%. The changes in the sensor impedance increase as the level of strain applied increases. This can be understood due to the decrease of the sensor capacitance as the distance between interdigitated fingers increases upon stretching. By comparing the relative change in the measured capacitance of the sensor with the strain, the gauge factor (GF) of the sensor was found to be approximately 0.47. It was reported that the resistance between two touching graphene flakes (out-of-plane resistance), which is a function of size, thickness, morphology, and distance between graphene flakes, plays more significant role in determining the sensitivity of graphene-based strain sensor [48]. Understanding this, it can therefore be implied that an improvement in GF of the proposed sensor can be expected by applying a proper pressure during the transfer of graphene to Kapton, to the point where the porosity of the graphene network is compensated at a minimum level. Another possible solution we consider in the future to improve further the GF would be to insert bipolar surfactant molecules (e.g., sodium deoxycholate (SDOC)) in between graphene flakes to increase their interlayer distance and hence the out-of-plane resistance as demonstrated in [48].

**Figure 13:**
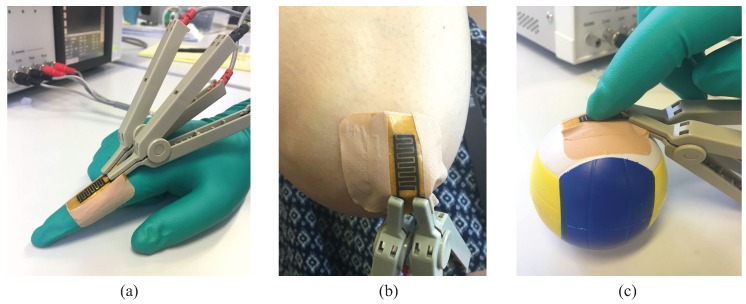
Implementation of the sensor for real-time strain sensing. The sensor was attached to the (**a**) finger and (**b**) elbow joints of a 36-year-old volunteer for repetitive movements monitoring. (**c**) The sensor was attached to a ball for tactile sensing scenario.

The setup for the human body-induced strains sensing are given in Figure 13. The first scenario was to apply the sensor to sense the repetitive movements of two selected body joints, i.e., finger and elbow joints (Figure 13a,b), while the second scenario was to apply the sensor to sense the repetitive touching of a gloved index finger (Figure 13c). In the first scenario, the sensor was attached directly on the corresponding joints of a 36-year-old female volunteer with adhesive tape, and the impedance response of the sensor was measured while the volunteer was performing repetitive movements of her finger and elbow. The sensor was positioned on the body in such a way that the applied strain mainly influenced the main sensing area of the sensor. In the second scenario, the sensor was attached over a soft surface of a 3 cm-radius ball with tape, and the impedance response was measured while the repetitive touches were applied by the volunteer. In all testing scenarios in this section, an AC signal of 1 V RMS with 1000 Hz frequency was applied to the electrodes. During the experiments, the volunteer was instructed to keep the movements as consistent as possible. Particularly for the second scenario, the touches were maintained to be on the same spot of the sensor without any significant pressure.

**Figure 14:**
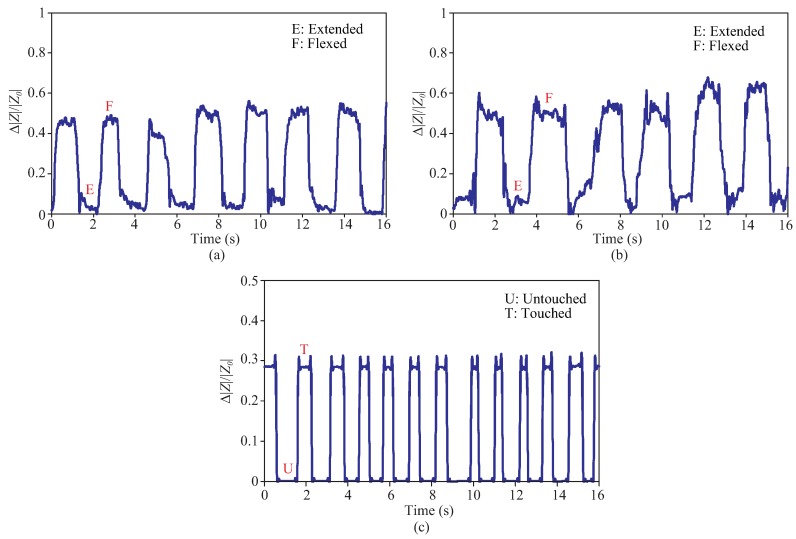
Responses of the sensor with respect to (**a**) repetitive movements of finger joint, (**b**) repetitive movements of elbow joint, and (**c**) repetitive gently touches of index finger.

The responses of the sensor towards the first sensing scenario are shown Figure 14a,b. The terms ‘Extended (E)’ and ‘Flexed (F)’ refer to the states where the volunteer was asked to extend and flex her joint, respectively. The change in the sensor impedance (|ΔZ|) is shown relative to the impedance of the initial position (|$Z_{0}$|), i.e., when the finger/elbow joint was extended. It can be seen from the results that the sensor was successful in reflecting the movements of the finger and elbow joints. The impedance variations are in sync with the change of strain induced to the sensor when the joints switch from one state to another. A slightly higher change of the impedance was observed in the case of the elbow which suggests a slightly higher strain induced to the sensor by this joint. Small aberrations which are present in each state indicates the natural variations occurred in each movement done by the volunteer.

The response of the sensor for the tactile sensing is shown in Figure 14c, where the terms ‘Untouched (U)’ and ‘Touched (T)’ pertain to the non-contact and contact states of the volunteer’s index finger and the sensor. The change in the sensor impedance (|ΔZ|) is shown relative to the initial impedance of the sensor (|$Z_{0}$|), i.e., ‘Untouched (U)’ state. A stable impedance variation corresponding to the changes between two states is shown. It was observed during the experiment that the sensor impedance changed promptly without any delay once touched gently by the gloved finger. This suggests that the change was predominantly induced by the permittivity change of the surrounding material (i.e., the presence of the finger on the surface of the sensor), rather than the pressure-induced mechanical deformation and recovery of the sensor. In some cases, the latter mechanism results in more response delay due to the intrinsic slow resilience processes of typical synthetic polymer [5,7]. When the gloved finger, which is a grounded conducting medium, touched the sensor, the electromagnetic couplings between interdigitated electrodes were affected. This led to the decrease in the sensor capacitance and hence the increase in the sensor impedance [7,11]. The effectiveness of the tactile sensing of this capacitive sensor is also attributed to the prominent fringing fields of a coplanar type capacitive sensor as explained before.

**Figure 15:**
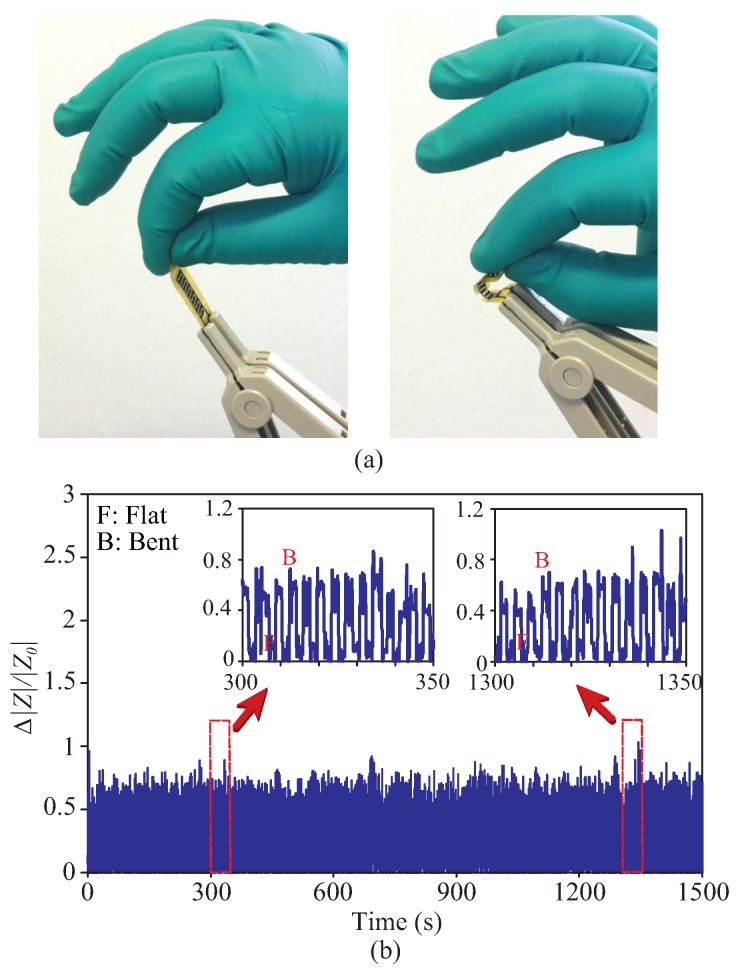
(**a**) Illustration of manual bending for investigating long-term response of the sensor (left: flat, right: bent). (**b**) Response of the sensor towards consecutive oscillatory bending for a duration of 1500 s. The inset of the figure shows the two separate time frames considered to validate the consistency in their responses.

Understanding the importance of the sensor durability in strain-sensing applications, we have also conducted a long-term mechanical test. The repetitive bending-releasing cycle with approximately 10 mm bending radius was applied manually by hand to the sensor as illustrated in Figure 15a. The duration of each test was 25 min long and the process was repeated three times giving a total of approximately 1300 bending-releasing cycles. The division of the experiment into three separate phases was due to the manual setup of the test, where it was very challenging to maintain the consistency of the applied strain or bending curvature for a long period of time. The sensor performance from one of the testing cycles is given in Figure 15b, showing a stable long-term response towards the change in its physical structure. As before, the change in the sensor impedance (|ΔZ|) is shown relative to the impedance when the sensor is in flat position (|$Z_{0}$|). The similar response was observed in two other testing phases with a variation in the change of impedance not exceeding 10%. This variation can be understood mainly due to the unavoidable deviation in the bending curvature applied manually by the volunteer’s hand. The accuracy and resolution of the proposed sensors were found to be approximately 2.3% and 3.8% of the full scale, respectively.

## 5. Conclusions

In this paper, we have presented the implementation of laser-induced graphene for realizing a flexible sensor for multiple sensing applications. The photo-thermal induction of polyimide film to generate the graphene and its subsequent use as electrodes on a flexible substrate allow for a low-cost, easy, yet effective realization of graphene-based sensor. The applications of developed sensor for various sensing purposes have also been demonstrated successfully. Firstly, we employed the sensor for electrochemically sensing the difference of sulfate concentrations in water samples. Secondly, the same sensor was employed for monitoring of the repetitive movements of elbow and finger joints as well as finger touches of a human subject. The sensitive responses of the sensor towards the given stimuli suggest the great feasibility of the proposed approach to realize a multifunctional flexible sensor. The performance of the sensor, however, can be improved further, for instance, by introducing selectivity on the electrodes towards specific ions through molecular imprinting polymer, as we demonstrated in [49,50]. This is to improve the electrochemical sensing sensitivity of the sensor, of which the effectiveness will be validated through the introduction of various interfering ions in the solution, as in the case of real chemical sensing applications. Another plan would be the inclusion of bipolar surfactant molecules [48] to improve the out-of-plane resistance of the graphene network and hence the sensor GF. These all possibilities are considered promising to be conducted as future work, together with the investigations on the effects of these methods on multi-functionality of the sensor. It is also planned to investigate further in the future the long-term durability of the sensor through more repetition and longer duration of both electrochemical and mechanical tests.
